# Facilitators and barriers to compliance with a raw coal ban amongst pregnant women and mothers of young children in Ulaanbaatar, Mongolia: A mixed-methods study

**DOI:** 10.1371/journal.pone.0323149

**Published:** 2026-04-03

**Authors:** Rob Miller, Emma Dickinson-Craig, Sophie Harbach, Charles Vickers, Enkhdulguun Amgalan, Mark Baker, for the MNUMS APF Group, Ninjbadgar Zorigtbaatar, Terkhen Turbat, Mandukhai Ganbat, Chimedsuren Ochir, David Warburton, G. Neil Thomas, Semira Manaseki-Holland, Jargalsaikhan Badarch, Rosie Day

**Affiliations:** 1 Institute for Applied Health Research, University of Birmingham, Birmingham, United Kingdom; 2 Mongolian National University of Medical Sciences, Ulaanbaatar, Mongolia; 3 Department of Epidemiology and Biostatistics, School of Public Health, Mongolian National University of Medical Sciences, Ulaanbaatar, Mongolia; 4 The Saban Research Institute, Children’s Hospital Los Angeles, Keck School of Medicine, University of Southern California, Los Angeles, California, United States of America; 5 School of Geography, Earth and Environmental Sciences, University of Birmingham, Birmingham, United Kingdom; Onom Foundation, MONGOLIA

## Abstract

Ulaanbaatar, Mongolia, is in the midst of a winter air pollution crisis driven by the combustion of solid fuels in the peri-urban Ger districts to which young children, fetuses and pregnant women are particularly vulnerable. To address this, the Mongolian government banned the sale and use of raw coal in May 2019 and has subsidized and promoted refined coal briquettes as an alternative fuel. This mixed-methods study utilized semi-structured interviews (n = 30) and a questionnaire survey (n = 369) to identify facilitators and barriers to compliance with the ban amongst Mongolian mothers living in Ulaanbaatar. Four main themes were identified that affected compliance: knowledge about air pollution, information sources, initial policy impacts and governance. Facilitators of participants’ ban compliance included awareness of the severity of air pollution’s health impacts, support for the policy and governmental enforcement. Concerns regarding fuel alternative safety and affordability, as well as uncertainty about the policy itself (inadequate information) were found to be barriers. Incorporation of these insights in future air pollution mitigation strategies could be beneficial for strengthening their effectiveness, both in Mongolia and settings that face similar challenges throughout the world, which in turn could be a crucial step in meeting the 2030 Sustainable Development Goals agenda.

## Introduction

Mongolia’s capital, Ulaanbaatar, experiences severe winter air pollution primarily due to the burning of raw coal for cooking and heating in rudimentary households in the so-called ‘Ger districts’ around the city center. These Ger districts contain Gers (traditional Mongolian tent-like structures) and small brick houses that often lack formal infrastructure. Due to high rates of rural to urban migration in recent years, Ulaanbaatar is now home to almost half of Mongolia’s population and due to lack of affordable housing options, permanent Ger districts have arisen around the city center [1]. During winter months, temperatures in Mongolia can fall as low as −40 degrees Celsius and for Ger district residents, the primary means of staying warm is by using traditional stoves in which, until recently, they burned raw coal [2,3]. There are more than 200,000 Ger district households in Ulaanbaatar, which in the past have been estimated to burn more than 1 million tons of raw coal throughout winter [4].

Raw coal combustion releases significant levels of pollutants into the city, via stove chimneys which transport smoke outside of the dwelling. Of particular concern is particulate matter with diameters equal to or less than 2.5 microns (PM_2.5_), widely considered one of the most harmful pollutants [5]. Wintertime ambient PM_2.5_ in Ulaanbaatar is on average 15 times the WHO recommended health-based guideline of 5 μg/m^3^ [6]. With these persistently high levels of exposure, it is therefore not surprising that, in 2016, the World Health Organization (WHO) attributed 1800 deaths to indoor air pollution and 1500 deaths to outdoor air pollution, in Mongolia [1]. Women, fetuses and children are particularly vulnerable. Lower respiratory tract infections have been linked to exposure to air pollution and are a leading cause of disease and disability in children under 5 years old in Mongolia [7–9]. Similarly, studies have linked asthma rates and decreased child lung capacity to air pollution exposure in Ulaanbaatar [2,10]. Moreover, adverse pregnancy outcomes associated with air pollution exposure include low birth weight, preterm birth, stillbirth and spontaneous abortion [11,12] These pollution-related health consequences place a heavy burden on the healthcare system. Indeed, a UNICEF report stated that the health costs of Mongolian air pollution-related disease in children alone will rise by 33% from 2017 to 2025, resulting in an increase of 4.8 billion Mongolian Tugrik (approx. 1.6 million dollars) per year, if significant action is not taken [13]. A more recent report estimated ambient air pollution to result in more than 2,800 deaths and approximately US$369 million in economic losses annually, while household air pollution contributes to an estimated 4,350 deaths and US$1.2 billion (MNT 3.9 trillion) in economic losses each year [14].

Multiple attempts to address air pollution have been made in Ulaanbaatar and the issue has been gaining attention in recent years. The long-term effectiveness of past air pollution interventions (APIs), such as the introduction of subsidized improved stoves, has been disappointing; a qualitative study conducted in Ulaanbaatar in 2004 suggested this was mainly due to poor adoption and weak governance [15]. That survey identified a lack of knowledge about the improved stove interventions, and potential associated health benefits, to be the leading barriers to stove-use behavior change. Lessons learned from such API investigations should be key for future API policies in Mongolia.

In May 2019 athe Mongolian government introducedthe Raw Coal Ban (RCB), which aimed to reduce air pollution by banning the distribution and use of raw coal for domestic and small businesses in the city of Ulaanbaatar and main surrounding districts. As an alternative, the government has been promoting the use of coal briquettes through subsidizing their price and heavy investment in their production [3]. Assessments since its introduction have shown improvements in key air pollutants [16,17]. However, the long-term health benefits of air pollution interventions are dependent on sustained compliance. The effectiveness of air pollution mitigation strategies relies not only on their implementation but also on continued public involvement and cooperation. Pregnant women and mothers of young children living in the highly polluted Ger districts surrounding the city were identified as key beneficiaries of such interventions, given both their own and their children’s heightened vulnerability to the health impacts of air pollution, as well as women’s (traditionally) central role in household stove management. It was deemed highly relevant to investigate the factors influencing uptake and compliance with the air pollution intervention among this population group. This study therefore aimed to identify the barriers and facilitators to compliance with the RCB for pregnant women and mothers living in Ger district households in Ulaanbaatar. In turn, findings of this study aim to inform and improve adoption of future air pollution mitigation policies in Mongolia as well as setting facing similar air pollution challenges throughout the world.

## Materials and methods

### Study design

The study was conducted in the first year after implementation of the RCB (February – June 2020) and utilized a mix-method study design, comprising semi-structured interviews and questionnaires that were piloted prior to the study.

### Setting, participants and data collection

Women who were currently pregnant (any trimester) and/or women with at least one child aged five or under, who resided in the major Ger districts of Ulaanbaatar, and who used a solid fuel stove for heating and/or cooking were included in this study. Women under 18 years of age, those who lacked capacity to consent, or who for any reason could not actively participate in discussion were excluded from participation. Recruitment of participants from major Ger districts in Ulaanbaatar took place in family health centers and district hospitals during attendance for routine check-ups.

For the semi-structured interviews, a minimum of 30 participants, including at least 15 mothers and 15 pregnant women, were targeted as sufficient to achieve data saturation [18]. Because of the significant overlap between these two groups, they were not treated separately in data collection and analysis. Face to face recruitment using purposive sampling by local medical staff initially occurred for one week at a district hospital in Chingeltei Ger district. Snowballing from initial recruits, where participants recommended individuals they thought would be interested in the study and who fit the study criteria, was then utilized. All interviews were conducted between February and March 2020 in Mongolian via telephone, due to COVID-19 restrictions, by a trained local interviewer. This form of communication introduced limitations, such as the inability to interpret body language, but was necessary in the interests of public health. Participants received reimbursements for their contribution in the form of phone credit. Demographics information was collected to obtain sense of study population characteristics.

Both in parallel and sequential to the interviews, quantitative data was collected using face-to-face questionnaires at a medical facility setting. Women attending antenatal check-ups or other routine doctors’ appointments in the five major Ger districts (Bayanzürkh, Byangol, Chingeltei, Songino Khairkhan, and Sükhbaatar) were recruited by fieldworkers from the Mongolian National University of Medical Sciences using convenience sampling and an opt-in approach. A target sample size of 400 women was calculated The required sample size was calculated using the standard formula for estimating a single population proportion: n = Z² × p(1 − p)/ d², where n is the required sample size, Z is the Z-score corresponding to the desired confidence level, p is the expected population proportion, and d is the desired margin of error [19]. An expected proportion of support of 60% (p = 0.60) was assumed based on prior evidence from the Asia Sustainable and Alternative Energy Program [20]. A 95% confidence level was selected (Z = 1.96), with a margin of error of ±5% (d = 0.05). Applying these values into the formula yielded a minimum required sample size of 369 participants. To account for potential non-response or incomplete questionnaires, a target sample size of 400 women was set. The questionnaire contained 5 sections: i) demographic characteristics, ii) the participant’s knowledge about the severity of air pollution and its health effects, iii) their stove and fuel use, iv) their opinions of air pollution reducing strategies and v) the perceived facilitators and barriers that influenced their implementation of these strategies. The questionnaire contained Likert scales (0–5 and 0–10) to assess knowlegde, support and perceived air quality before and after the ban.

### Data analysis

#### Qualitative analysis.

The audio-recordings were transcribed verbatim in Mongolian before the transcripts were translated into English. The research assistants who carried out these tasks were given training and a sample of translations was checked by a second translator to ensure accuracy in words and meaning. Data from the first interviews were analyzed before later interviews were carried out, allowing the research tools, such as the topic guide, to be iteratively adapted and particular lines of enquiry to be pursued as the study progressed [21]. This also allowed the practice of reflexivity to be applied, as the positionality of the research team members was evaluated [22]. A constant comparative method was employed in order to perform a comprehensive and critical thematic analysis and to assess for saturation [23]. The data were coded and the Braun and Clarke’s six step approach was used to generate themes and organise ideas [24].

#### Quantitative analysis.

Responses were compiled in an Microsoft Excel spreadsheet and descriptive statistics (frequencies, means and standard deviations) were used to summarize demographics and outcome variables. Variables measured using the Likert Scale were categorized where appropriate to ensure adequate expected counts per group for Pearson’s chi-square testing and to reflect the distinction identified in the qualitative findings (e.g., low, moderate, and high levels of support). All categorization decisions were finalised prior to statistical testing. Chi-squared tests were performed to assess differences between these categorical variables in contingency tables (S1 Dataset. Air pollution questionnaire.). P-values of less than 0.05 were considered statistically significant and all tests were two-tailed. All data were analyzed using Stata/SE 16.1 and Prism GraphPad.

### Ethics

This study received ethical approval from the University of Birmingham’s Internal Research Ethics Committee [IREC2019/1636480 and IREC2019/1646103] and the Mongolian National University of Medical Sciences Review Board [#2019/3–12]. Permission to conduct research was also received from Ulaanbaatar’s Health Department and from each district health center used as a recruitment site. All participants gave fully-informed consent, either written or verbal. Once the Covid-19 pandemic made it unsafe to gain written consent in-person in some circumstances, the ethics review committee approved gaining fully-informed consent via telephone, using the same consent items as the written consent form, and with this verbal consent being witnessed by the researcher, audio-recorded and transcribed as part of the interviews.

## Results

A total of 30 interviews and 369 questionnaires were completed amongst pregnant women and mothers in Ulaanbaatar between February and June 2020.

Demographic information obtained during the semi-structures interviews reveiled the majority of participants resided in the Songino khairkhan, Bayanzhurk, Chingeltei and Khan-Uul districts, were aged 20–35 and the majority were married. Employment status that was recorded showed that most women were employed. Almost half was pregnant during the interviews, whilst the rest had a child between 0–5 years old. Parity ranged from 0 to 4 children (average 2). All variables had missing data for a number of participants. Demographics from the questionnaire participants (quantitative study) are shown in Table 1.

**Table 1:** Study population demographic of quantitative study participants.

Variable	Amount (%)
**District**	135 (36.6)
Songino Khairkhan	
Chingeltei	66 (17.9)
Sükhbaatar	119 (32.2)
Bayanzürkh	18 (4.9)
Byangol	31 (8.4)
**Age**	
<20	2 (0.5)
20-34	261 (70.7)
≥35	104 (28.2)3 (0.8)
Blank	2 (0.5)
**Marital status**	
Married	351 (95.1)
Not married	18 (4.9)
**Level of education**	
Did not finish high school	1 (0.3)
High school diploma	139 (37.7)
College/ vocational degree	38 (10.3)
Bachelor’s degree (4 years)	157 (42.5)
Graduate degree (PhD)	16 (4.3)
Still studying	18 (4.9)
**Employment**	
Employed	246 (66.7)
Unemployed	27 (7.3)
Student	96 (26.0)
**Household income**	
Less than 400.000₮	21 (5.7)
400.000₮ - 900.000₮	183 (49.6)
Over 900.000₮	150 (40.6)
Rather not say	15 (4.1)

After analyzing both sets of data, four main themes were identified that affected compliance with the ban amongst mothers: ‘Knowledge about air pollution’, ‘Information sources’, ‘Initial RCB policy impacts’ and ‘Governance of RCB implementation’.

Below we present qualitative and quantitative findings for each theme, of which key facilitators and barriers are summarized in Table 2.

**Table 2:** Overview of identified facilitators and barriers to adoption of air pollution interventions by mothers in Ulaanbaatar, Mongolia.

Key facilitators and barriers to maternal air pollution intervention adoption	
Facilitators	High awareness of: severity of the ambient air pollution problem; causes of air pollution; short-term health consequences from air pollution exposure (e.g., respiratory symptoms)
	Perceived effects of the intervention on air quality (visual and physical)
	Availability of information sources on the intervention
	Strong governmental implementation and legal enforcement of the intervention
	Sense of responsibility to engage with the intervention
Barriers	Low awareness of: health consequences of indoor air pollution exposure; long-term health consequences from air pollution expsure; vulnerability of pregnant women and children
	Informal sources of information regarding air pollution and health
	Perceived inadquacy of information given about the intervention (quantity and detail)
	Perceived ineffectiveness of fuel alternative (i.e., heat produced, length of burning time, wood requirement for lighting)
	Safety concerns of fuel alternative (i.e., CO poisoning and exploding stoves)
	Concerns over cost of fuel alternative (instead of raw coal)
	Concerns over transport costs and logistics (more frequent fuel collection)
	Poverty and insufficient accessability of fuel alternative

### Knowledge about air pollution

All qualitative interviewees were aware of air pollution in Ulaanbaatar and stressed its high levels, while some stated the problem has become much worse in recent years.

“Air pollution was okay when I first moved here… It has increased dramatically in the last ten years” – Interviewee 10

“it was 2007 when air pollution didn’t exist” – Interviewee 16

Air pollution was heavily associated with place and interviewees distinguished between ambient and indoor air pollution. Two women believed indoor air pollution was less of a risk because it could be reduced by cleaning.

“Inside, outside? Totally different. I think outdoor pollution is greater and inside air pollution is okay, because we clean it” – Interviewee 14

When asked to rate indoor and ambient air pollution severity in the questionnaire (0 being no pollution, 10 worst pollution), responses echoed the interview findings that air pollution was considered high, particularly outdoors: 54% (n = 199) of respondents indicated ambient air pollution was severe (rated ≥7/10), compared to only 26% (n = 97) giving such high ratings for the air pollution inside their homes.

The Ger districts were perceived to be heaviest polluted, while other areas of Ulaanbaatar were believed to be little affected. The views of many interviewees present a perceived dichotomy between those living in Ger households and other accommodation; Ger households were associated with the burning of fuels and air pollution while others were seen to represent a higher standard of living. Many interviewees named the burning of solid fuels in Ger districts, vehicles and high population-density as causes of air pollution.

“if you’re not living in an apartment, generally it’s kind of hard [to reduce air pollution], as long as you live in a Ger, you will burn coal” – Interviewee 9

In the questionnaire, women were asked to choose the main air pollutant sources in Ulaanbaatar using a multi-response question. Almost all, (n = 338, 92%) mentioned residential fuel burning as a pollution source, of whom 43% (n = 157) indicated this being the sole contributor to Ulaanbaatar’s air pollution problem. Vehicle use, powerplants, dust and waste were amongst the other sources identified as causes of Ulaanbaatar’s air pollution.

Many interviewees appeared to base their understanding of the severity of air pollution on physical sensation, remarking that smoke obscured visibility and pollution had a strong odor. The belief that air pollution had reduced this year, since introduction of the RCB, was, consequently, often based on personal observations of improved visibility and reduced smell. To some interviewees, physical perception was such an important gauge of the problem that they remarked that support for an intervention would depend on whether it reduced the sensation of air pollution.

“It’s ok to ban raw coal. If this refined coal produces less smoke and smells less” – Interviewee 14

All interviewees understood air pollution to have a negative health impact, with most emphasizing its severity. When asked how they believed air pollution affects health, almost all interviewees spoke of respiratory symptoms, in both children and adults, such as coughs and sore throats. For many this appeared to be based on personal experience and the knowledge that air and consequently air pollution enters the human body via the lungs.

“I think it is really bad for our lungs. There are the symptoms like coughing, so it is harmful for our pulmonary system” – Interviewee 1

“because of the smoke, all of our tissue and organs are getting sick via the lungs” – Interviewee 6

While most interviewees mentioned the short-term health effects of air pollution exposure such as respiratory symptoms, specific diseases linked to longer term exposure such as lung cancer were only mentioned by a few people.

“lung cancer cases are increasing these days, I think it is related to air pollution” – Interviewee 19

Similarly in the questionnaire, when presented with a list of various health problems and asked which were thought to be caused by air pollution, the commonest problems identified were: coughs 324 (88%), shortness of breath 259 (70%) and pneumonia 259 (70%).

The majority of interviewees were aware that pregnant women and children are at risk when exposed to air pollution, and that air pollution can cause negative pregnancy outcomes such as miscarriage.

“it was believed that being the height of an adult dog increased exposure to air pollution, and so small children are exposed to air pollution because of their similar height. So it probably affected children a lot” – Interviewee 29

“suddenly my doctor said “Your child’s heart has stopped” (during pregnancy). I found out it was because of the air pollution.” – Interviewee 3

Several interviewees remarked that they or their children would stay indoors to reduce air pollution exposure. Older people were also often identified as vulnerable. The strength of someone’s immune system, their comorbidities and their height were seen, by some, to be related to their health risk.

When questionnaire participants were asked to list which population groups were at risk when exposed to air pollution, 39% (n = 143) identified pregnant women, 42% (n = 153) babies and 35% (n = 128) children and as a risk group. When asked how much they thought air pollution affected their own child’s/children’s health, rated out of 10 (1 being not affected and 10 very severely affected) the average rating was 7.9 (SD 2.6).

### Information sources

Interviewees received information on air pollution (covering air pollution levels, health impacts, interventions etc.) from various sources such as televised news, social media websites, leaflets, advertising boards at bus stations and word of mouth. Opinions on the quality and quantity of this information, however, were very mixed. Some believed there was enough relevant information available while others stated the opposite: that information was insufficient and lacking in detail, especially concerning air pollution health effects.

“It is not enough. Even if people talk about it… they don’t talk about it in detail… and it seems that kind of information is rare… Everyone knows a bigger view instead of knowing details” – Interviewee 14

In the questionnaire, the majority of women evaluated their own level of knowledge about air pollution and its associated health effects as ‘average’ n = 151 (41%) or ‘above average’ n = 139 (38%).

When asked through a multi-response question which sources participants used to get information about air pollution, 205 (56%) mentioned social media, 186 (51%) newspapers, 138 (38%) the national meteorology agency (NAMEM) and 130 (35%) governmental outlets such as leaflets and public announcements. Fourty percent (n = 148) of all respondends indicated to only use non-official sources, such as social media, newspapers and friends. Within thisgroup, those who got information from family and friends, either exclusively or in combination with other sources, had the lowest self-rated knowledge, with only 18 (28%) of them regarding their knowledge as ‘above average’. Within the group respondends that indicated to use at least some for our official information, (60%, n = 148). Those who (also) used academic literature or information from doctors, had the highest self-reported knowledge, with 57% (n = 13) and 44% (n = 25) respectively, regarding their knowledge as ‘above average’. Those who received governmental information on air pollution predominantly rated their knowledge as ‘average’ (n = 55, 42%). Nevertheless, the majority of participants (n = 292; 79%) agreed that governmental distribution of air pollution information to the public would be an effective measure to improve air quality.

### Initial RCB policy impacts

#### Perceptions of the effectiveness of the RCB.

While the majority of interviewees were supportive of the RCB and had experienced improved air quality since its inception, there were differing opinions on its health benefits.

“there was a lot of air pollution before we started using the refined fuel… children got sick more often and there was increased preterm birth because of this” – Interviewee 21

“In appearance air pollution looks like it has decreased and the smoke is much less. But in terms of its impact on the human body, it is slightly higher than in previous years” – Interviewee 26

The questionnaire recorded a marked increase in support of a RCB since earlier measures, with 75% (n = 276) of participants being supportive of the RCB, compared to 60% of households in 2007 agreeing that burning raw coal should be banned [14]. When assessing the perceived improvements in air quality, the large majority of questionnaire participants (90%) found air pollution levels to have improved since the last, pre-ban winter (2018/19), although many still viewed it as a serious problem. In 2007, 85% of participants believed burning of raw coal to be the main source of air pollution, compared to 92% in our study [14].

Many interviewees said that they were wearing facemasks, but due to the COVID-19 pandemic and not to prevent air pollution exposure. Furthermore, some observed that while the incidence of respiratory infection appeared to have decreased, this could have been due to COVID-19 measures such as mask wearing and increased hygiene awareness, rather than the RCB.

“Nowadays, people wear facemasks, sanitize their hands and the schools and kindergartens are closed so catching colds is reducing” – Interviewee 5

When assessing women’s perceptions of ambient air pollution in the Ger district since the introduction of the RCB, 90% (n = 331) of all respondents found that air pollution had reduced (Table 3). There was a significant decrease in the number of women who rated the air pollution as severe (>6/10) in the winter following the introduction of the RCB (2019/2020) compared to the winter before, 54% (n = 199) versus 91% (n = 337) respectively (χ^2^ (1)=132.6 p < 0.001). Respondents’ support for the ban was indicated using a scale from 0 to 10 (0 being no support and 10 being full support). 75% (n = 276) were shown to be in favor of the ban (support rated as >6/10) and 41% (n = 152) rated their support as 10 out of 10. A smaller proportion (n = 24, 6.5%) of respondents gave their support of the ban as low (<4/10) or middling (n = 69, 19% 4–6/10). A large majority (n = 336, 91%) of those who supported the ban, believed that air pollution had improved since the introduction of the policy. Consequently, perceived improvement of air quality since introduction of the RCB was found to be associated with its support χ^2^ (2)=6.08 p = 0.048.

**Table 3:** Descriptive statistics of perceived air quality in the Ger districts and UB city center, as well as self-rated knowledge per subcategory.

Variable	Subcategories	Frequency (%)
Perceived ambient air pollution city center winter 2019/2020 (post RCB)	Low (<4/10)	54 (15)
	Moderate (3–6)	179 (49)
	Severe (>6/10)	136 (37)
	*Overall median (IQR)*	*6(4-8)*
Perceived ambient air pollution city center winter 2018/2019 (pre RCB)	Low (<4/10)	12 (3)
	Moderate (3–6)	39 (11)
	Severe (>6/10)	318 (86)
	*Overall median (IQR)*	*8 (7-10)*
Perceived ambient air pollution Ger district winter 2019/2020	Low (<4/10)	31 (8)
	Moderate (3–6)	138 (38)
	Severe (>6/10)	199 (54)
	*Overall median (IQR)*	*7(5-9)*
Perceived ambient air pollution Ger district winter 2018/2019	Low (<4/10)	16 (4)
	Moderate (3–6)	14 (4)
	Severe (>6/10)	337 (91)
	*Overall median (IQR)*	*10(9-10)*
Self-rated knowledge on air pollution	Below average (<3/5)	79 (21)
	Average (3/5)	151 (41)
	Above average (>3/5)	139 (38)
	*Overall median (IQR)*	3 (3-4)
Support for the RCB policy	Low supportive (<4/10)	24 (7)
	Moderately supportive (3–6)	69 (19)
	Highly supportive (>6/10)	276 (75)
	*Overall median (IQR)*	9 (6-10)

Practical considerations included the effectiveness of the alternative briquette fuel for heating and cooking; however, views on this were conflicting. Some stated that briquettes burn for longer and with greater heat, producing less smoke compared to the raw coal they used before. Others in contrast stated that briquettes emit less heat and need to be burnt more frequently. Many found using refined briquettes required more wood for (re)lighting and some said this is because briquettes are harder to light.

“The refined briquette doesn’t burn on its embers and requires more effort… The previous fossil fuel didn’t burn like that” – Interviewee 4

Similarly, questionnaire responses reflected an inconsistent understanding as to how briquettes burn and whether they are more or less effective than coal, with nearly equal amounts of respondents agreeing (40%) or disagreeing (41%) that briquettes burn quicker. Meanwhile, relatively more respondents found briquettes to be heat efficient (48% disagreed vs 28% agreed that they produce less heat).

#### Unintended consequences.

Many interviewees expressed safety concerns regarding the use of refined briquettes. Some had heard stories of briquettes causing stoves to explode while many were concerned by the possible association between refined briquettes and deaths due to carbon monoxide (CO) poisoning. This had led to some viewing briquettes as dangerous and, in some cases, undermined support for the RCB.

“We didn’t suffer from carbon monoxide because of the old fuel, but we do because of this new fuel. Therefore, it is not that good” – Interviewee 22

Some interviewees, however, remarked that poisoning was due to burning briquettes in an unsafe way, such as not ventilating stoves, not cleaning stoves regularly enough or burning too many briquettes at once. Some said that instructions on burning the briquettes correctly and safely were available, but two interviewees said that these only became available after people started to suffer from CO poisoning. Similarly, multiple interviewees talk of receiving CO alarms from the government but some remark that this only occurred later on in winter.

“the official instructions [on briquettes] started to be given to us after people started suffering due to carbon monoxide” – Interviewee 20

27% (n = 99) of participants agreed/strongly agreed that briquettes are dangerous and have other health effects that coal does not have. 16% (n = 59) agreed/strongly agreed that briquettes create more smoke than coal. Only a small minority of women (6%) were aware of the government providing carbon monoxide alarms. When asked about the potential effectiveness of providing such alarms, 17% of those who responded felt they would be extremely effective.

### Governance of RCB implementation

Despite the varied perceptions on the effectiveness of the RCB, the interviewees remarked that ultimately, they had to comply with the RCB because its legal framework removed choice and meant raw coal was no longer on sale.

“We are forced to buy it [refined briquettes]… If they sold the fossil fuel [raw coal], I would buy it because it heats well” – Interviewee 5

Many interviewees believed, however, that this legal barrier was not entirely effective. They believed some people in the Ger districts were still burning banned fuels such as raw coal and other materials prohibited for combustion under the 2016 waste ban.

“what they use is fuels and if they can’t find any, they just burn anything like old clothes, or tires, in order not to freeze” – Interviewee 6

Many interviewees were unsure how the RCB was being enforced and there was disparity in experience of enforcement; some said police or other authority figures had visited their homes to check their fuel use, while others said no such visit had occurred. No qualitative interviewees admitted to having breached the laws themselves. All interviewees who were asked whether they had any leftover raw coal from the previous winter replied that they did not, some explaining that they believed raw coal can lose its heating ability over time.

In the quantitative questionnaire, 288 (78%) stated they currently used briquettes as their primary heating source, compared to only 12% (n = 45) in winter before the ban. While only 32 (9%) said that they could still purchase raw coal on the black market, only 5 (1%) of participants admitted they still used raw coal for either cooking or heating since the ban was introduced and they all also burnt either wood or briquettes alongside coal. 119 (32%) believed the police would not find out if they burned raw coal, whilst 116 (31%) believed the police would find out, showing a lack of consensus as to whether policing of the ban is effective and universal enough to ensure people do not try and return to using coal if they wish.

Several interviewees did not only feel legally compelled to engage with the ban but also thought individuals had a collective responsibility to comply with government measures, in order for them to be maximally effective.

“the government can’t tackle it [air pollution] alone and individuals also can’t tackle it alone” – Interviewee 12

“The government has to regulate and we have to follow these regulations, it’s like a collaboration”- Interviewee 2

Restrictions on household briquette purchase quantities were implemented by the government in order to ensure enough was available throughout the winter while production was still being upscaled. Many interviewees said that the government restricting each household to buying six sacks of refined briquettes per week meant they had insufficient fuel supplies. One interviewee even said this had been a relatively warm winter, suggesting the problem would be exacerbated by colder weather (when winters can reach -40C). Conversely, one said people had spent more time than usual at home that winter due to measures against COVID-19, such as the closing of schools and certain workplaces, meaning more fuel may have been burnt than usual. Another interviewee believed that insufficient briquette supplies had led to profiteering schemes.

“I heard some people talking and they said that sometimes they couldn’t find this refined coal… And people are selling it… for profit” – Interviewee 10

Some interviewees said that a weekly limit on briquette purchases has made more frequent fuel pickups necessary because previously, raw coal supplies could be bought to last a number of weeks. This caused difficulties and potentially further expenses in transporting fuel back to the household.

“It’s easy for those who have a car. Sometimes when I look around, elderly people are pushing wheelbarrows to get briquettes. People can’t catch a taxi to buy only one or two briquettes… So, it seems bad in all respects” – Interviewee 26

Many also complained that the briquettes themselves were more expensive than coal. One interviewee believed that the poorest Ger residents were being forced to burn substances other than refined briquettes in order to survive the harsh UB winter (e.g., garbage waste, car tires). Potentially, exposure to these fumes could have worse health consequences.

“some people, who are impoverished, burn their clothes” – Interviewee 9

In the questionnaire, 51% (n = 189) agreed/strongly agreed that briquettes are easy to find and buy. 31% (n = 116) reported worries that the RCB would cause financial problems due to the cost. 57% of participants in the low-income bracket expressed financial worries about the cost of alternative fuels, compared to only 30% in the high-income bracket. This suggests that household financial status and poverty is a barrier to engagement with the RCB.

## Discussion

This study aimed to identify key facilitators and barriers to compliance with an air pollution intervention for a vulnerable group of population (pregnancy women and mothers of young children) in one of the world’s most renowned polluted cities, Ulaanbaatar. Despite a decade of attemps to reduce its winter air pollution problem, Mongolia has faced mulitple challenges in API adoption and sustainable effectiveness [25]. A 2011 World Bank analysis of air pollutants in Ulaanbaatar showed that, without decisive intervention, associated health problems will increase in severity and prevalence [26]. An estimated 10% of deaths in Ulaanbaatar are attributable to air pollution, which violates the right to live in a healthy environment, as set out in the Constitution of Mongolia [3]. Implementation and widespread adoption of effective air pollution mitigation strategies in Ulaanbaatar is therefore essential to protect its population’s health. Click or tap here to enter text.

When assessing the perceived improvements in air quality, the large majority of questionnaire participants (n = 311, 90%) thought that air pollution levels had improved since last winter, although many still viewed it as a serious problem. In a 2007 survey conducted by the Asia Sustainable and Alternative Energy Program, 85% of participants thought that burning of raw coal was the main source of air pollution in Ulaanbaatar, compared to 92% in our study [20].

The strong implementation of the ban by the Mongolian government (in a country accustomed to high levels of government intervention), including removing raw coal from the market, banning its use as well as subsidizing and heavily investing in briquettes, is evidently a facilitator of engagement.

In this study, many participants reported that obtaining sufficient quantities of briquettes—combined with the need for more frequent travel to purchase fuel, as briquettes were perceived to burn more quickly than raw coal—resulted in higher overall costs. These challenges reflect broader issues of energy poverty, defined as the inability to secure adequate household energy services without financial hardship. Previous evidence demonstrates that fuel poverty is closely linked to adverse health outcomes, as households facing high energy costs may be forced to make trade-offs between heating, nutrition, and other essential needs (Liddell & Morris, 2010). The burden of fuel costs is not distributed equally: the World Bank’s 2016 ‘Clean Stove Initiative’ found that low-income Ger households spent up to 31% of their income on fuel, compared with only 6% among the highest-income households [27]. Consequently, without targeted financial protections, fuel transition policies risk exacerbating existing socioeconomic and health inequalities.

Poverty and a lack of financial resources can prevent compliance to pollution-reducing measures [28]. In this study, many participants reported that obtaining sufficient quantities of briquettes each time combined with the need for more frequent travel to purchase fuel, as briquettes were perceived to burn more quickly than raw coal, resulted in higher overall costs. These challenges reflect broader issues of energy poverty, defined as the inability to secure adequate household energy services without financial hardship. Previous evidence demonstrates that fuel poverty is closely linked to adverse health outcomes, as households facing high energy costs may be forced to make trade-offs between heating, nutrition, and other essential needs [27]. The cost of fuel disproportionately affected low-income households, as shown by the World Bank’s 2016 report on the ‘Clean Stove Initiative’, which found that Ger households with the lowest incomes spent 31% of their income on fuel, compared to the highest income households, which spent just 6% of their income on fuel [29].

Other factors which may have impacted on the participants’ complaince and support to the RCB included how easy or difficult it was to obtain briquettes and whether burning briquettes was perceived as being dangerous. At the time of the study, accessibility of briquettes was perceived to be low due to government limits on quantities per household, regardless of household size emphasising inequality in fuel access [30]. Briquette shortages were a concern, due to limited briquette manufacturing in the single factory in Ulaanbaatar, leading to rationing of fuel per household. Since then, however, both briquette availability and accessibility have increased. An additional briquette factory was put into operation in late 2020 and distribution centers were put in close proximity to the Ger districts.

The API’s perceptible impacts on air pollution, such as reduced smoke and odor, was identified as a potential facilitator of participant support and engagement. This resonates with previous research, which stresses the significant impact an individual’s perception of air pollution in their local environment has on their understanding of air pollution and its risks [31,32]. Other reasons for increased support for the RCB is also a potential facilitator, which could be the result of increased knowledge of air pollution, increased levels of air pollution overthe past decade and perceived improvements in air quality and healthsince its introduction.

Most of these factors can be addressed by improving public education on general air pollution and information on the API itself. Efforts made to improve accessibility of information about the target of the API, its expected (evidence-based) effects on air quality and health, as well as information on actual effectiveness during/since its implementation could be key to long-term adherence. Addressing safety concerns is also crucial, as previous research shows fears over an API’s safety can reduce willingness to change behavior [33].

The results of the questionnaire should be viewed in the light of the methodological limitations and the concurrent COVID-19 pandemic. All participants were women and women often express higher levels of concern about potential health risks, reducing the generalizability (to men) of the findings [34]. However, we believe the findings can be generalizable to the households in ger districts. Traditionally, there has been a gendered division of responsibility in Mongolian households, in which men were responsible for the livestock management and women for the household tasks, including maintaining the stove. Whilst these responsibilities have shifted to some extent once herding families moved into the city, also accompanied by a steep rise in literacy (>97%) and employment rates amongst women, taking care of the children and keeping the household warm is still mostly the woman’s task. Furthermore, this study could be affected by social desirability bias due to the unlikeliness of participants admitting to breaking laws such as the RCB, particularly when being recorded and interviewed by university researchers who could potentially have been seen as agents of the establishment [35]. While this may have toned down the reports of adherence to RCB, we believe it would not not affect the other responses to questions.

Due to the first winter following the ban coinciding with the early stages of the pandemic, certain perceived barriers to adoption of the briquette fuel alternative might have been the result of the pandemic measures rather than the RCB policy itself. For example, public health measures relating to the COVID-19 pandemic such as frequent hand washing, facemask wearing and social distancing may have affected health perceptions and responses. It was also not possible to carry out focus group discussions during the outbreak, but instead we believe the individual interviews provided an equally good or better in-depth understanding of participants’ perceptions during the crucial early stages of the policy implementation.

Despite the limitations of the pandemic, gaining timely insights on mothers’ perceptions and fuel use behavior following the Ulaanbaatar RCB, has been crucial to gain more understanding of facilitators and barriers to air pollution intervention compliance amongst this most vulnerable and susceptible groups, potentially relevant to other interventions being developed in Mongolia (e.g., UNICEF CHIPS program), or in similar settings in Central Asia or other polluted cities [36]. These insights can play a crucial part when interpreting findings on the effectiveness of the RCB or other interventions in terms of public adoption as well as its effects on air quality and health. Click or tap here to enter text. Improving the air quality can thuss accelerate efforts to meet the sustainable development goals (SDG) 11 (sustainable cities and communities) and SDG 3 (health) for all residents, and particular vulnerable groups such as pregant women and children. In order to continue progress towards such goals, effective solutions must be sought to improve air quality for all, which can only become sustainable if findings such as ours related to facilitators and barriers to adoption are taken into account.

## Conclusions

This mixed-methods study in Ulaanbaatar identified four main themes (knowledge about air pollution, information sources, initial policy impacts and governance) that affected compliance with the latest API amongst the vulnerable mothers and pregnant women. Awareness of the severity of air pollution’s health impact amongst mothers was generally high, trusted information was sourced by many from official, academic and medical sources. The ban was fairly quickly seen to have some impact, and overall the legal enforcement of the ban had prevented many participants from burning raw coal. These factors were found to be facilitators of intervention adoption. That the health impacts of indoor air pollution were perceived by some to be low, use of informal sources of information, concerns regarding refined coal briquette safety, and cost implications leading to financial difficulties were found to be potential barriers. Integrating such insights into future air pollution interventions could increase their long-term adoption and overall effectiveness, which could be a crucial step in meeting the 2030 SDG agenda in Mongolia and similar settings.

### Institutional review board statement

This study was conducted in accordance with the Declaration of Helsinki. The research was approved by the BMedSci Population Sciences and Humanities Internal Ethics Review Committee of the University of Birmingham (IREC2019/1636480 and IREC2019/1646103).

### Informed consent statement

All participants gave fully-informed consent, either written or verbal.

## Supporting information

S1 Dataset Air pollution questionnaire. (XLSX)

## References

[1] Air pollution in Mongolia. Bull World Health Organ. 2019;97(2):79–80. doi: 10.2471/BLT.19.020219 30728613 PMC6357570

[2] Dashdendev B, Fukushima LK, Woo MS, Ganbaatar E, Warburton D. Carbon monoxide pollution and lung function in urban compared with rural Mongolian children. Respirology. 2011;16(4):653–8. doi: 10.1111/j.1440-1843.2011.01958.x 21362106

[3] Government of Mongolia. Mongolia voluntary national review 2019 implementation of the sustainable development goals. Government of Mongolia. 2019. www.nda.gov.mn

[4] Raw coal consumption to be banned. Montsame. https://montsame.mn/en/read/133813. 2018. Accessed 2022 October 16.

[5] Ambient (outdoor) air pollution. World Health Organisation. https://www.who.int/en/news-room/fact-sheets/detail/ambient-(outdoor)-air-quality-and-health. 2021. Accessed 2022 October 16.

[6] Allen RW, Gombojav E, Barkhasragchaa B, Byambaa T, Lkhasuren O, Amram O, et al. An assessment of air pollution and its attributable mortality in Ulaanbaatar, Mongolia. Air Qual Atmos Health. 2013;6(1):137–50. doi: 10.1007/s11869-011-0154-3 23450113 PMC3578716

[7] Jadambaa A, Spickett J, Badrakh B, Norman RE. The impact of the environment on health in Mongolia. Asia Pac J Public Health. 2014;27(1):45–75. doi: 10.1177/1010539514545648 25113526

[8] Woolley KE, Bartington SE, Kabera T, Lao X-Q, Pope FD, Greenfield SM, et al. Comparison of respiratory health impacts associated with wood and charcoal biomass fuels: a population-based analysis of 475,000 children from 30 low- and middle-income countries. Int J Environ Res Public Health. 2021;18(17):9305. doi: 10.3390/ijerph18179305 34501907 PMC8431364

[9] Al-Janabi Z, Woolley KE, Thomas GN, Bartington SE. A Cross-Sectional Analysis of the Association between Domestic Cooking Energy Source Type and Respiratory Infections among Children Aged under Five Years: Evidence from Demographic and Household Surveys in 37 Low-Middle Income Countries. Int J Environ Res Public Health. 2021;18(16):8516. doi: 10.3390/ijerph18168516 34444264 PMC8394069

[10] Yoshihara S, Munkhbayarlakh S, Makino S, Ito C, Logii N, Dashdemberel S, et al. Prevalence of childhood asthma in Ulaanbaatar, Mongolia in 2009. Allergol Int. 2016;65(1):62–7. doi: 10.1016/j.alit.2015.07.009 26666488

[11] Amegah AK, Quansah R, Jaakkola JJK. Household air pollution from solid fuel use and risk of adverse pregnancy outcomes: a systematic review and meta-analysis of the empirical evidence. PLoS One. 2014;9(12):e113920. doi: 10.1371/journal.pone.0113920 25463771 PMC4252082

[12] Enkhmaa D, Warburton N, Javzandulam B, Uyanga J, Khishigsuren Y, Lodoysamba S, et al. Seasonal ambient air pollution correlates strongly with spontaneous abortion in Mongolia. BMC Pregnancy Childbirth. 2014;14:146. doi: 10.1186/1471-2393-14-146 24758249 PMC4024019

[13] UNICEF. Mongolia’s air pollution crisis: A call to action to protect children’s health. 2018. https://www.unicef.org/mongolia/reports/call-action-protect-childrens-health

[14] Leopold C, Hagens A, Dai J, Tordrup D, Dolgorsuren S, Grafton D, et al. Investment Case for Air Pollution Reduction in Mongolia. UNDP; 2024. https://research-portal.uu.nl/en/publications/investment-case-for-air-pollution-reduction-in-mongolia/

[15] Gordon JK, Emmel ND, Manaseki S, Chambers J. Perceptions of the health effects of stoves in Mongolia. J Health Organ Manag. 2007;21(6):580–7. doi: 10.1108/14777260710834364 18062610

[16] Dickinson-Craig E, Turbat T, Hemming K, Pope FD, Bartington SE, Anjaa S. Improving air quality and childhood respiratory health in Mongolia: the impact of the raw coal ban and COVID-19 restrictions—an interrupted time-series analysis. Atmosphere. 2025;16(1):46. doi: 10.3390/ATMOS16010046

[17] Dickinson-Craig E, Bartington SE, Watts R, Mandakhbayar O, Khurelbaatar E-O, Ochir C, et al. Carbon monoxide levels in households using coal-briquette fuelled stoves exceed WHO air quality guidelines in Ulaanbaatar, Mongolia. Int J Environ Health Res. 2023;33(12):1760–71. doi: 10.1080/09603123.2022.2123906 36206479

[18] Saunders B, Sim J, Kingstone T, Baker S, Waterfield J, Bartlam B, et al. Saturation in qualitative research: exploring its conceptualization and operationalization. Qual Quant. 2018;52(4):1893–907. doi: 10.1007/s11135-017-0574-8 29937585 PMC5993836

[19] Pourhoseingholi MA, Vahedi M, Rahimzadeh M. Sample size calculation in medical studies. Gastroenterol Hepatol Bed Bench. 2013;6(1):14–7. 24834239 PMC4017493

[20] Asia Sustainable and Alternative Energy Program ASTAE. Mongolia energy efficient and cleaner heating in poor, peri-urban areas of Ulaanbaatar summary report on activities funded by the Asia Sustainable and Alternative Energy Program. 2008.

[21] Pope C, Ziebland S, Mays N. Qualitative research in health care. Analysing qualitative data. BMJ. 2000;320(7227):114–6. doi: 10.1136/bmj.320.7227.114 10625273 PMC1117368

[22] Dodgson JE. Reflexivity in Qualitative Research. J Hum Lact. 2019;35(2):220–2. doi: 10.1177/0890334419830990 30849272

[23] Silverman D. Doing qualitative research. 4th ed. Los Angeles, USA: SAGE. 2013.

[24] Braun V, Clarke V. Using thematic analysis in psychology. Qual Res Psychol. 2006;3:77–101. doi: 10.1191/1478088706qp063oa

[25] Puzzolo E, Stanistreet D, Pope D, Bruce N, Rehfuess E. Factors influencing the large-scale uptake by households of cleaner and more efficient household energy technologies Systematic review i) Factors influencing the large-scale uptake by households of cleaner and more efficient household energy technologies. London: EPPI-Centre; 2013. http://eppi.ioe.ac.uk/

[26] The World Bank. M O N G O L I A Air Quality Analysis of Ulaanbaatar Improving Air Quality to Reduce Health Impacts Environment and Social Development. 2011. www.worldbank.eapenvironment

[27] Liddell C, Morris C. Fuel poverty and human health: a review of recent evidence. Energy Policy. 2010;38(6):2987–97. doi: 10.1016/j.enpol.2010.01.037

[28] Laferriere KA, Crighton EJ, Baxter J, Lemyre L, Masuda JR, Ursitti F. Examining inequities in children’s environmental health: results of a survey on the risk perceptions and protective actions of new mothers. Journal of Risk Research. 2014;19(3):271–87. doi: 10.1080/13669877.2014.961518

[29] The World Bank. The Cost of Air Pollution Strengthening the Economic Case for Action. 2016.

[30] Jun S. Is the raw coal ban a silver bullet to solving air pollution in Mongolia?: a study of the Mongolian government’s air pollution reduction policies and recommendations in the context of COVID-19. Journal of Public and International Affairs. 2021.

[31] Bickerstaff K, Walker G. Public understandings of air pollution: the ‘localisation’ of environmental risk. Global Environmental Change. 2001;11(2):133–45. doi: 10.1016/s0959-3780(00)00063-7

[32] Day RJ. Traffic-related air pollution and perceived health risk: lay assessment of an everyday hazard. Health, Risk & Society. 2006;8(3):305–22. doi: 10.1080/13698570600871869

[33] Astuti SP, Day R, Emery SB. A successful fuel transition? Regulatory instruments, markets, and social acceptance in the adoption of modern LPG cooking devices in Indonesia. Energy Research & Social Science. 2019;58:101248. doi: 10.1016/j.erss.2019.101248

[34] Reames TG, Bravo MA. People, place and pollution: investigating relationships between air quality perceptions, health concerns, exposure, and individual- and area-level characteristics. Environ Int. 2019;122:244–55. doi: 10.1016/j.envint.2018.11.013 30449629

[35] Lavrakas PJ. Social desirability. Encyclopedia of survey research methods. Thousand Oaks, California, United States of America: Sage Publications, Inc. 2008. doi: 10.4135/9781412963947.n537

[36] Braham WW, Hakkarainen M, Buyan M, Janjindorj G, Turner J, Erdenekhuyag S. Cooking, Heating, Insulating Products and Services (CHIPS) for Mongolian Ger: Reducing Energy, Cost, and Indoor Air Pollution. Energy for Sustainable Development. 2022;71:462–79. doi: 10.1016/J.ESD.2022.10.017

